# Salt sensitivity potentiates high-salt diet-induced intestinal barrier disruption and gut microbiome dysbiosis in rats

**DOI:** 10.3389/fmicb.2025.1718782

**Published:** 2026-01-09

**Authors:** Baihan Zeng, Xile Peng, Pengyang Xiao, Kaidi Nie, Guilong Zhang, Lina Xia

**Affiliations:** School of Health Preservation and Rehabilitation, Chengdu University of Traditional Chinese Medicine, Chengdu, China

**Keywords:** high salt diet, salt sensitivity, gut microbiota, metagenomics, intestinal barrier disruption

## Abstract

**Introduction:**

The high-salt diet is a prevalent eating habit associated with health risks. This study investigated the impact of high salt on intestinal barrier disruption and gut microbiome dysbiosis using Wistar and Dahl salt-sensitive rat models.

**Methods:**

Rats were fed a normal diet or a high-salt diet for eight weeks. Body weight and plasma inflammatory cytokines were monitored in the study. Colon tissue damage was assessed via histopathological examination, and metagenomic sequencing was utilized to analyze alterations in microbial composition, functional pathways, and biodiversity.

**Results:**

The results indicated that high salt significantly elevated pro-inflammatory cytokine levels and induced structural damage in the colon. Metagenomic analysis revealed that high salt concentrations resulted in approximately a 15% difference in microbial species composition. And led to a decrease in Alpha diversity, along with an increase in the *Firmicutes/Bacteroidetes* ratio. Taxon-specific alterations included reduced abundance of *Lactobacillus* and *Clostridium*, and increased abundance of *Enterobacter* and *Bifidobacterium*. Correlation analyses further revealed a positive correlation between *Bifidobacterium* abundance and tumor necrosis factor-α level in Dahl salt-sensitive rats.

**Discussion:**

This study illuminates the gut microbiota’s role in salt-sensitivity and provides a foundational basis for developing microbiota-targeted interventions for at-risk individuals.

## 1 Introduction

The high-salt diet (HSD) is a common yet frequently overlooked unhealthy eating habit that leads to various chronic diseases (Kwong et al., 2023; Wang et al., 2025), such as hypertension, obesity, and multi-organ impairment (Abdelnour et al., 2020; Allen et al., 2019; Li et al., 2019). Given that the impacts of high salt intake may vary among individuals with different constitutions and likely involve multiple mechanisms, it is imperative to identify a universal physiological process that can explain the underlying mechanisms of HSD effects.

The gut harbors a vast array of microorganisms, predominantly bacteria, collectively referred to as the gut microbiota (Huang et al., 2024). These bacteria and their metabolites participate in regulating multiple physiological processes in the host (Li et al., 2023). Owing to the enormous number of genes encoded by the gut microbiota, it is often regarded as the second genome (Zhang et al., 2022; Feng et al., 2024). Recent studies indicate that gut microbial dysbiosis is a key mechanism in the pathogenesis of various disorders (Li et al., 2024a,b; Mu et al., 2024a,b; Wang et al., 2024; Mu et al., 2024a,b; Padhi et al., 2022). For instance, HSD modulates the abundance of *Lactobacillus* and *Bacteroides*, leading to elevated levels of pro-inflammatory T helper 17 (Th17) cells and enteric corticosterone, thereby contributing to hypertension and vascular pathology (Wilck et al., 2017a,b; Yan et al., 2020a,b; Monteleone et al., 2017). Additionally, high salt intake alters the abundance of *Proteobacteria* and *Bacteroidetes*, while increasing the levels of tumor necrosis factor-α (TNF-α) and interleukin-6 (IL-6), ultimately inducing inflammation (Guo et al., 2021; Li et al., 2020; Ma et al., 2020). The gut microbiota, along with its critical metabolites and signaling molecules, has recently garnered considerable attention across various branches of medicine (Li et al., 2024a,b; Miranda et al., 2018; Shen et al., 2021). It is considered a highly promising target for research, offering novel insights into the mechanisms underlying the effects of HSD (Xu et al., 2024). Therefore, utilizing animal models with well-defined genetic backgrounds to dissect the role of salt sensitivity in the HSD–gut microbiota–host health axis has important translational significance for identifying at-risk human populations and developing targeted microbiome-based intervention strategies.

**Figure 1:**
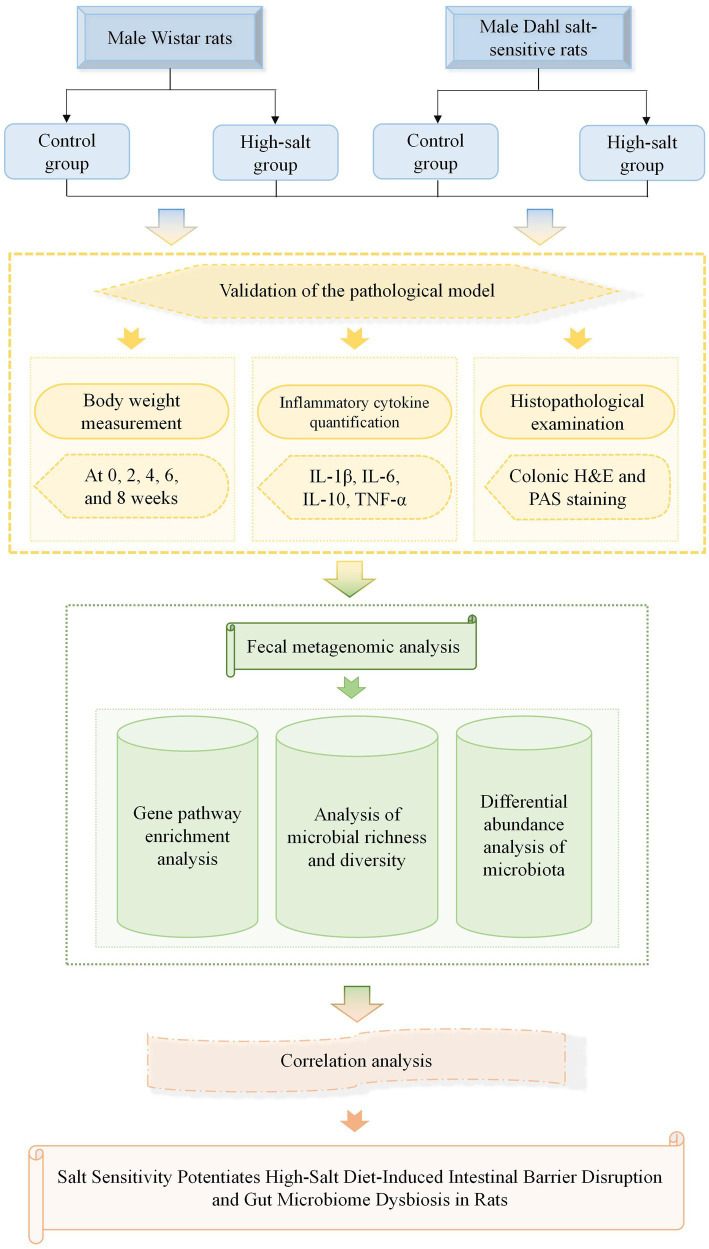
A schematic flowchart illustrating the overall experimental design and research procedures of this study.

In summary, this study builds upon previous research to confirm that HSD induces inflammatory injury and disrupts gut microbial ecology. In contrast to previous studies focusing on a single model, our study employs a parallel comparative design using both Wistar and Dahl salt-sensitive rats to decipher how host salt sensitivity dictates the gut microbiota’s response to HSD. Additionally, Further research investigates the mechanisms through which high salt intake affects the host via the gut microbiota. Wistar and Dahl salt-sensitive rats were selected as animal models for comparative analysis of salt-sensitivity-related damage. The Dahl salt-sensitive rat is widely recognized as one of the gold-standard models for studying salt-sensitive hypertension and end-organ damage in humans, given its strong pathophysiological resemblance to the human condition. Adverse physiological effects were assessed through changes in body weight and plasma inflammatory cytokine levels, while colon histopathology was used to evaluate structural damage to the intestinal tissue. Metagenomic sequencing of fecal samples was employed to detect enriched functional pathways and assess microbial richness and diversity. Correlation analysis was applied to explore the relationship between inflammatory damage and alterations in the gut microbiota. This study aims to provide a theoretical basis for the clinical prevention and mitigation of HSD-related health risks. The main schematic flowchart of this study is shown in Figure 1.

## 2 Materials and methods

### 2.1 Animals and diet

Wistar rats and Dahl salt-sensitive rats were obtained from SiPeiFu Biotechnology Co., Ltd. (Beijing, China). The rat feeds were purchased from KeaoXieli Feed Co., Ltd. (Beijing, China). To eliminate potential confounding effects of estrogen on body weight and other parameters, only male rats were used in this study. Animals (*n* = 12 DahL salt-sensitive rats, *n* = 12 Wistar) were housed under controlled conditions (22 ± 1 °C, 50–60% humidity, 12 h light/dark cycle) with ad libitum access to food and purified water. Following a one-week acclimatization period, Dahl salt-sensitive rats were randomly assigned to the normal control group (DC group, 0.3% NaCl diet feeding) or HSD group (DH group, 8% NaCl diet feeding). Similarly, Wistar rats were randomly assigned to the normal control group (WC group, 0.3% NaCl diet feeding) or HSD group (WH group, 8% NaCl diet feeding). The catalog numbers for the 0.3% NaCl diet and the 8% NaCl diet are 1,016,712,327,837,081,600 and 1,016,706,714,625,204,224, respectively. Diet compositions are detailed in Supplementary Table S1. Our animal study is reported in accordance with the ARRIVE (animal research: reporting of *in vivo* experiments) guidelines. All experimental procedures were approved by the Experimental Animal Ethics Committee of Chengdu University of Traditional Chinese Medicine (Ethics Approval No.: 2018-21).

### 2.2 Sample collection

At the end of acclimatization and weeks 2, 4, 6, and 8 post-modeling, body weights of all rats were measured using an electronic laboratory balance. Afterward, Rats were then anesthetized with 1% pentobarbital sodium (30 mg/kg) and euthanized. Blood samples (~10 mL/rat) were collected from the abdominal aorta, centrifuged at 3,000 × g for 10 min at 4 °C to obtain plasma, and stored at −80 °C. Plasma TNF-α, IL-6, interleukin-1β (IL-1β), and interleukin-10 (IL-10) levels were quantified using enzyme linked immunosorbent assay (ELISA) kits (Elabscience Biotechnology, Wuhan, China). Both the intra-assay and inter-assay coefficients of variation for all cytokine measurements were confirmed to be less than 10%. The specific sensitivity and detection range for each assay kit are provided in Supplementary Table S2. Following colon dissection with sterilized scissors, 2–3 fecal pellets were collected from the lumen using sterile forceps and aliquoted into 2-mL cryovials. Adjacent colon segments were harvested for histopathological analysis. All samples were stored at −80 °C until use.

### 2.3 Colon histopathological examination

The Colon samples were fixed in 10% neutral buffered formalin, processed routinely, embedded in paraffin, and sectioned at a thickness of 4 μm. Consecutive sections were subjected to either hematoxylin and eosin (H&E) staining for general histopathological evaluation or periodic acid-Schiff (PAS) staining for neutral mucins and basement membrane visualization. All stained sections were examined under an optical microscope. Representative images of colonic mucosa were captured at 200x magnification to assess overall tissue architecture (H&E) and mucin distribution/goblet cell abundance (PAS), and at 400× magnification to evaluate cellular details (H&E) as well as basement membrane integrity and detailed goblet cell morphology/mucin content (PAS).

### 2.4 Fecal metagenomic profiling

#### 2.4.1 DNA extraction

Genomic DNA was extracted from 0.25 g of thawed fecal sample using a commercial kit (QIAamp PowerFecal Pro DNA Kit, Qiagen, Germany). Samples were homogenized with solution C1, followed by a series of steps involving incubation with solutions C2 and C3, binding of DNA with solution C4, washing with solution C5, and final elution with solution C6. All centrifugation steps were performed at 10,000 × g at room temperature. The extracted DNA was stored at −20 °C for subsequent analysis.

#### 2.4.2 DNA shearing

A quantified amount of genomic DNA (as determined by Qubit fluorometer) was diluted to 100 μL with 1 × TE buffer (on ice). The DNA was mechanically sheared using a focused-ultrasonicator (Covaris, USA) according to the manufacturer’s recommendations to generate fragments of the desired size distribution. The fragmentation efficiency was verified by agarose gel electrophoresis.

#### 2.4.3 Purification of sheared products

The fragmented DNA was purified using AMPure XP beads (Beckman Coulter, USA) at a ratio of 1:1 (v/v). The mixture was incubated at room temperature for 5 min, separated on a magnetic stand, and the supernatant was discarded. The beads were washed twice with 80% ethanol, air-dried, and the DNA was eluted in 0.1 × TE buffer.

#### 2.4.4 End repair and adapter ligation

The purified DNA was subjected to end repair using a combination of buffer and end repair enzyme mix. The reaction was incubated in a thermal cycler. Subsequently, Illumina-compatible adapters and DNA ligase were added to the reaction mixture for adapter ligation under controlled conditions.

#### 2.4.5 Size selection and purification

Adapter-ligated DNA was purified and size-selected using a double-sided SPRI bead-based method (typically with 0.6 × and 0.15 × ratios of AMPure XP beads) to enrich for fragments of the desired length. The purified library was eluted in 0.1 × TE buffer.

#### 2.4.6 PCR amplification

The size-selected library was amplified by PCR using high-fidelity DNA polymerase and Illumina primer cocktails. The PCR conditions followed the manufacturer’s protocol.

#### 2.4.7 Purification of PCR products

The PCR amplicons were purified using AMPure XP beads (0.8 × ratio), washed twice with 80% ethanol, air-dried, and eluted in nuclease-free water. The final library was quantified and stored at −20 °C.

#### 2.4.8 Sequencing and primary bioinformatic processing

Sequencing was performed on an Illumina NovaSeq 6000 platform (Illumina, USA) to generate 150 bp paired-end reads. Raw sequencing data were processed as follows:

Quality control: Raw reads were quality-filtered and adapter-trimmed using Trimmomatic (v0.33) with default parameters. Host DNA Depletion: Reads aligning to the rat reference genome (mRn6) were identified and removed using Bowtie2 (v2.2.3). *De novo* Assembly: High-quality reads were *de novo* assembled into contigs using MEGAHIT (v1.1.2). Contigs shorter than 300 bp were discarded. Assembly quality was assessed using QUAST (v2.3). Gene Prediction: Open reading frames (ORFs) were predicted on the contigs using MetaGeneMark (v3.26). Non-redundant Gene Catalog Construction: Predicted protein sequences were clustered to remove redundancy using CD-HIT (v4.6.6) with parameters of 95% sequence identity and 90% coverage. The raw data, number of contigs assembled, and coverage for each sample are detailed in Supplementary Table S3.

#### 2.4.9 KEGG functional annotation

Protein sequences from the non-redundant gene catalog were aligned against the KEGG database (Release 105.0) using DIAMOND (v0.9.24) with a maximum *e*-value threshold of 1 × 10^−5^. The best hit for each sequence was retained for functional assignment.

#### 2.4.10 Functional diversity analysis

Based on the Kyoto Encyclopedia of Genes and Genomes (KEGG) annotation results, functional abundance profiles were generated. Subsequent analyses (e.g., PCA, differential abundance analysis) and visualization were performed using the BMK Cloud Metagenome Analysis system (Biomarker Technologies, China).

#### 2.4.11 Taxonomic diversity analysis

Taxonomic profiling was performed by aligning the non-redundant gene sequences against the NR database using DIAMOND. The relative abundance of taxa at different taxonomic ranks was calculated. Community composition and diversity analyses were conducted and visualized via the BMK Cloud system.

### 2.5 Correlation analysis

Correlation analyses and visualizations were performed using the data visualization module of the ‘Wu Kong’ platform (available at: https://www.omicsolution.com/wkomics/main/) (Zhou et al., 2023).

### 2.6 Statistical analysis

Data are presented as mean ± SD. Statistical analyses were performed using SPSS 26.0. Between-group differences were assessed by an independent t-test with *post hoc* comparisons (SNK for equal variances; Dunnett’s T3 for unequal variances). Significance was defined as *p* < 0.05.

## 3 Result

### 3.1 Differential body weight changes under HSD

**Figure 2:**
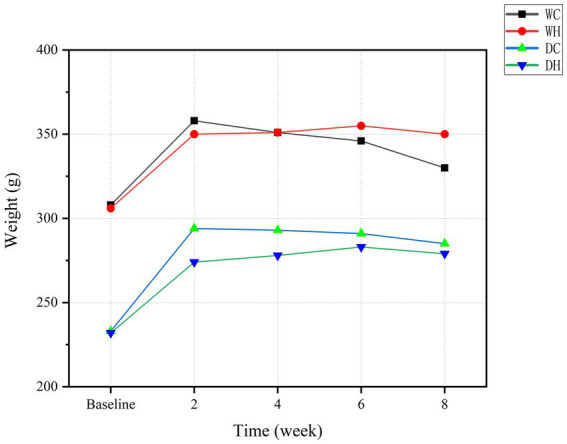
Changes in body weight of rats throughout the modeling period.

Body weight was measured at baseline (pre-intervention) and at weeks 2, 4, 6, and 8 during the modeling period, with specific values detailed in Supplementary Table S4. Longitudinal changes in body weight across groups are presented in Figure 2. During the initial 2 weeks, compared with the baseline level of this group, all groups exhibited significant weight gain, consistent with ongoing somatic development. Beyond this period, body weight plateaued or declined slightly, likely reflecting attenuated growth rates post-maturation.

Given inherent strain-dependent differences in body mass between Wistar and Dahl salt-sensitive rats, statistical comparisons were restricted within strains. At week 8, HSD increased the final body weight in Wistar rats, whereas it reduced weight in Dahl salt-sensitive rats. This difference response confirms systemic physiological alterations induced by HSD. Weight augmentation in the WH group may reflect hyperphagia secondary to sodium-enhanced palatability, whereas weight loss in the DH group suggests that salt sensitivity amplifies HSD-induced metabolic dysregulation. These metabolic perturbations prompted further investigation into intestinal barrier integrity and gut microbiota homeostasis.

### 3.2 HSD exacerbates systemic inflammation

**Figure 3:**
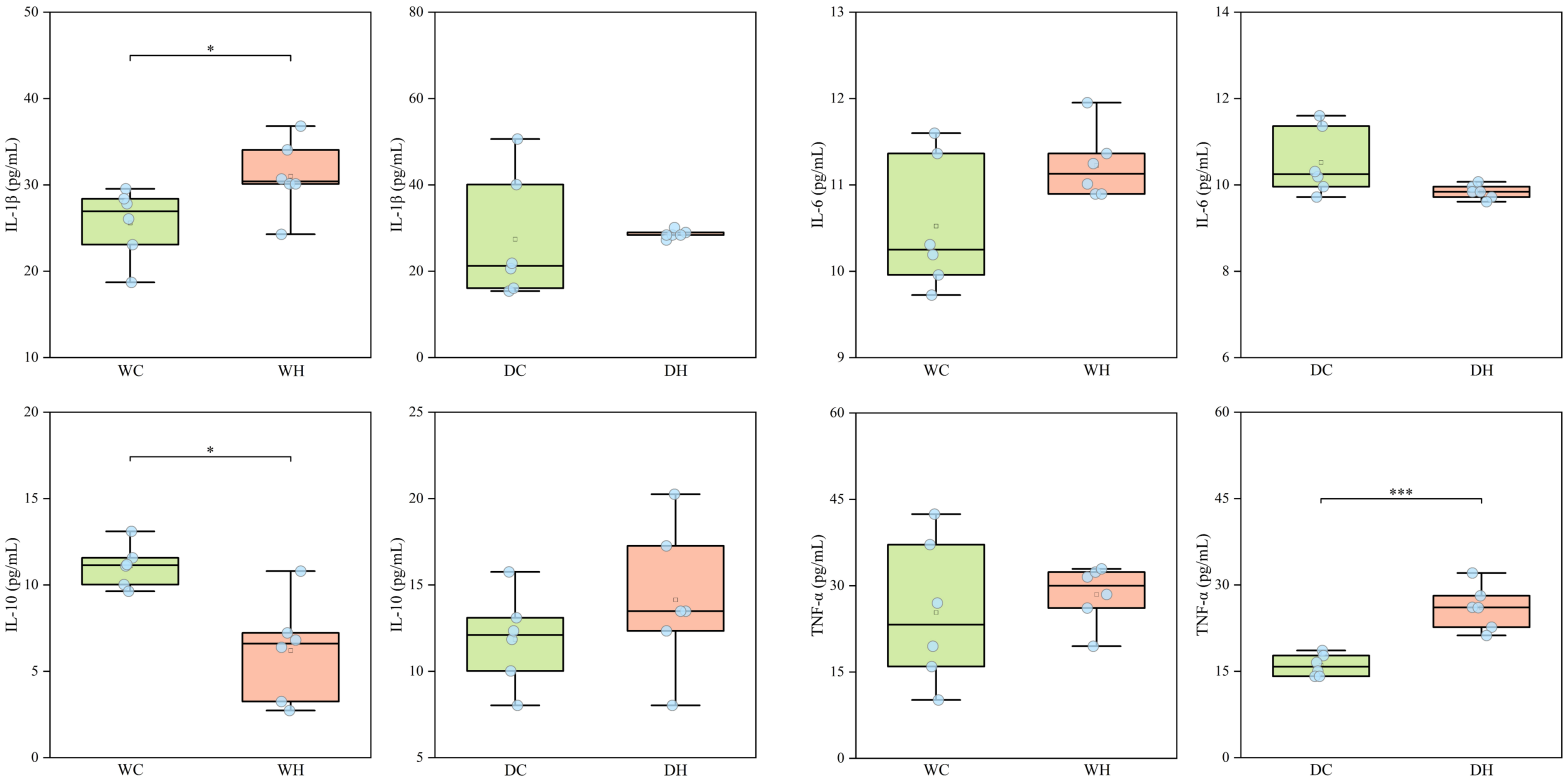
Inflammatory cytokine levels in rats (IL-1β, IL-6, IL-10, and TNF-α). *p < 0.05, **p < 0.01, ***p < 0.001.

To assess the impact of HSD-induced inflammation on intestinal integrity, plasma concentrations of inflammatory cytokines (including IL-1β, IL-6, IL-10, and TNF-α) were quantified across experimental groups. As depicted in Figure 3, these analyses revealed the implicated inflammatory pathways in HSD-associated pathophysiology. The specific values are detailed in Supplementary Table S5.

HSD elicited strain-divergent pro-inflammatory responses: In Wistar rats, HSD significantly elevated IL-1β levels and suppressed IL-10 (*p* < 0.05), with concomitant upward trends in IL-6 and TNF-α. This pattern implicates IL-1β/IL-10 axis dysregulation as the primary driver of HSD-induced inflammation in this strain. Conversely, salt-sensitive rats exhibited attenuated interleukin responses to HSD, but showed marked TNF-α hyperinduction (*p* < 0.001). This TNF-α-dominated inflammatory signature suggests distinct mechanistic pathways underlying salt-sensitivity-associated tissue injury.

### 3.3 HSD-induced colon tissue damage

**Figure 4:**
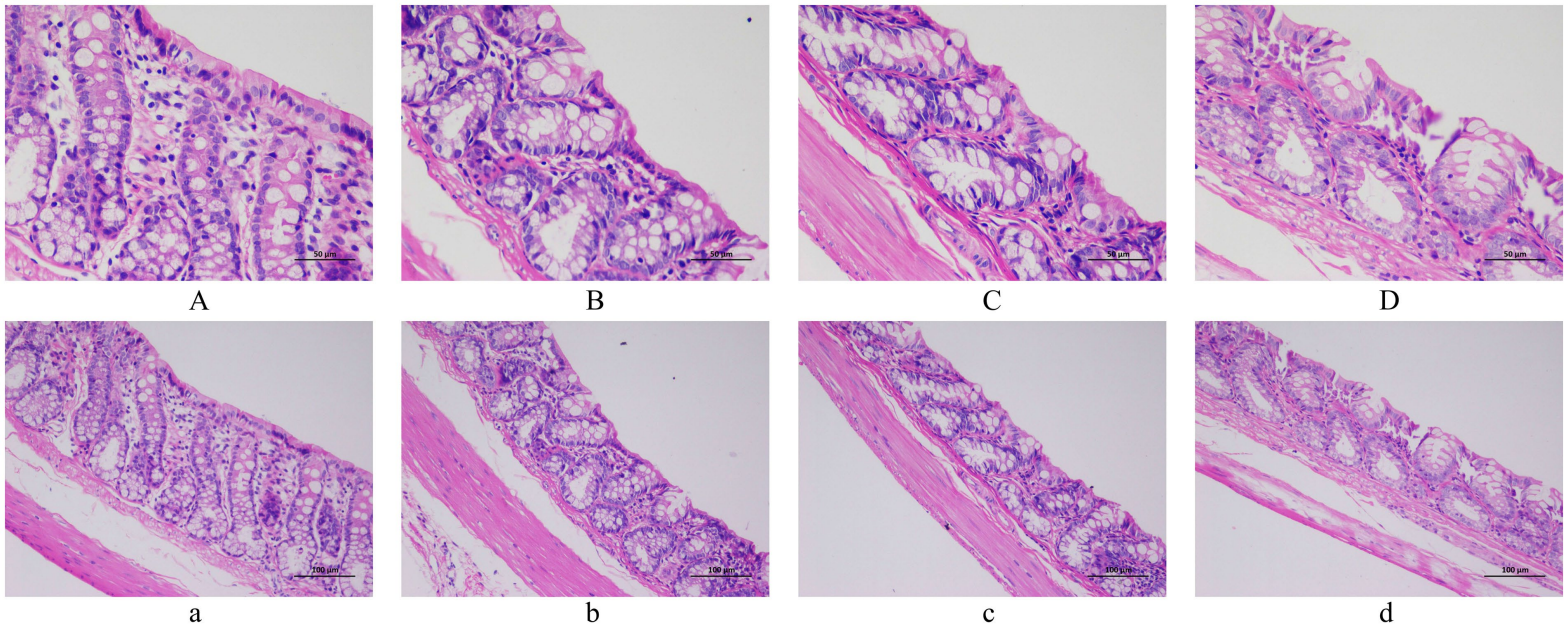
Histopathological analysis of colon tissues by H&E staining. **(A,** a**)** WC group. **(B,** b**)** WH group. **(C,** c**)** DC group. **(D,** d**)** DH group. Scale bars: 500 μm **(A–D)** and 100 μm (a–d).

**Figure 5:**
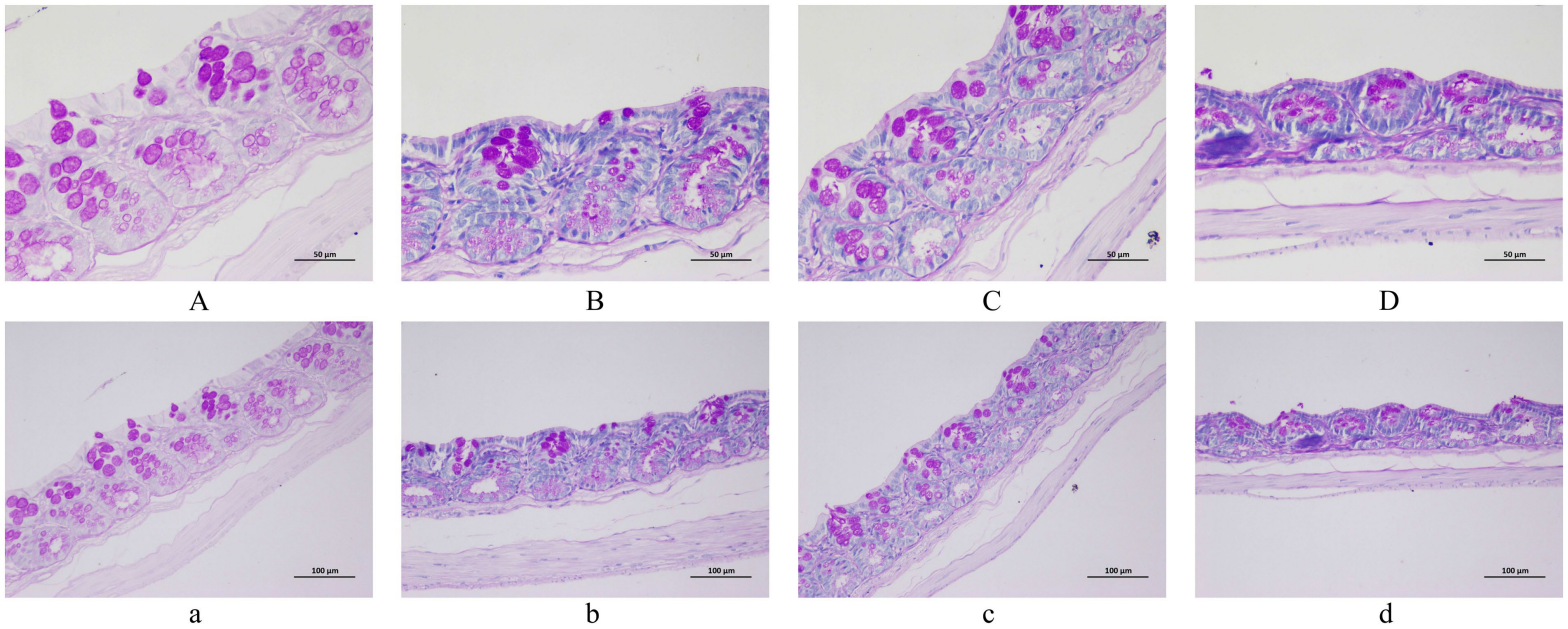
Histopathological analysis of colon tissues by PAS staining. **(A,** a**)** WC group. **(B,** b**)** WH group. **(C,** c**)** DC group. **(D,** d**)** DH group. Scale bars: 500 μm **(A–D)** and 100 μm (a–d).

Histopathological assessment of rat colon sections (H&E/PAS staining) demonstrated that HSD induced significant colonic damage. The colon pathological sections of 200x and 400x magnifications among all groups are displayed in Figures 4, 5. HE staining revealed dense inflammatory infiltrates, crypt distortion, epithelial ulceration, and cellular degeneration. PAS staining concurrently demonstrated goblet cell depletion, mucin hyposecretion, and focal basement membrane fragmentation across magnifications, indicating critical mucin-physical barrier compromise. Crucially, the DH group exhibited more severe inflammatory damage, establishing that salt sensitivity potentiates HSD-induced intestinal barrier disruption through aggravating epithelial damage.

### 3.4 Metagenomic profiling of gut microbiota

**Figure 6:**
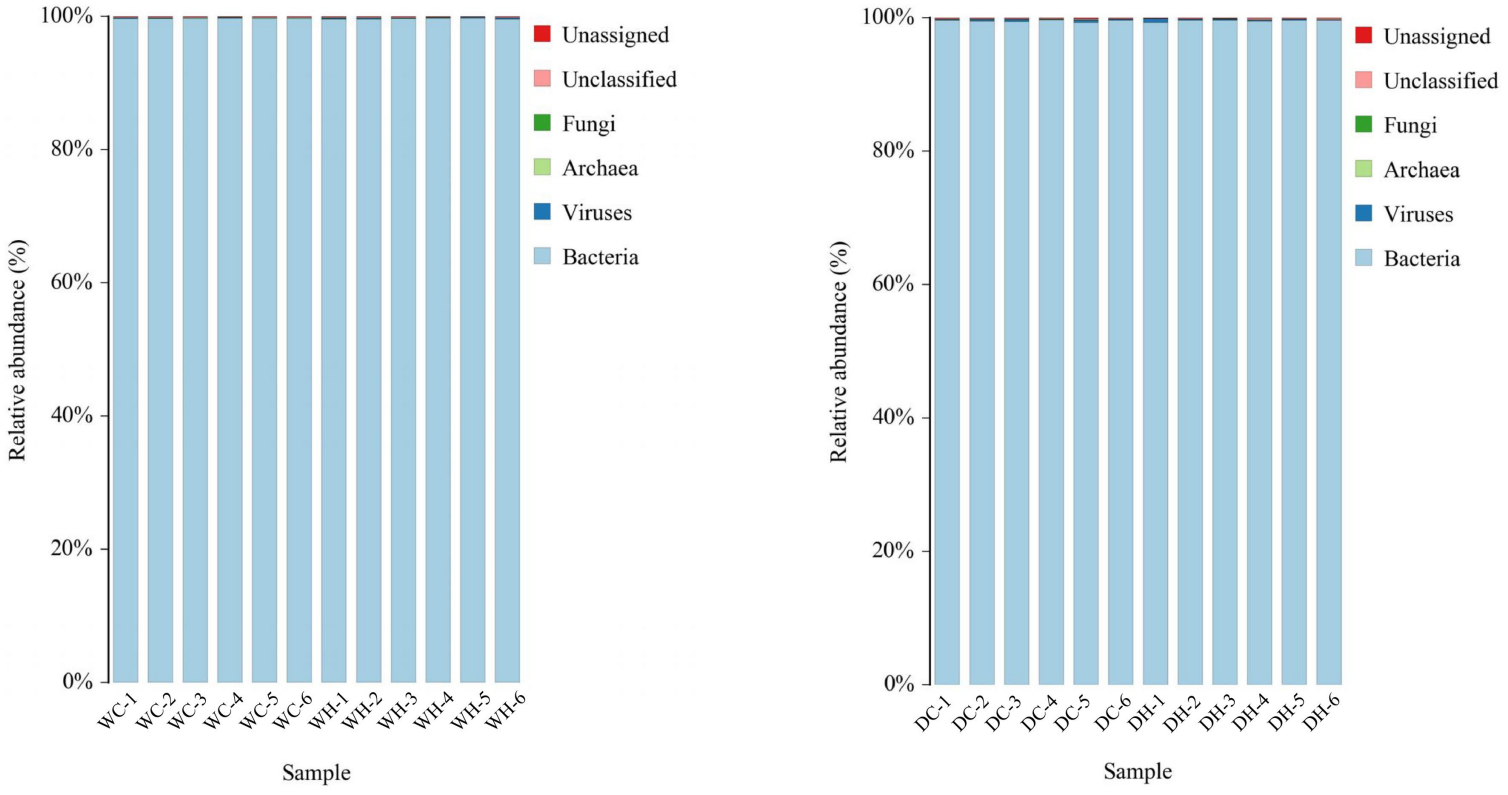
The species composition bar chart. The *x*-axis represents the sample name; the *y*-axis represents the relative abundance percentage. One color represents a species, and the length of the color block represents the relative abundance ratio of that species.

To accurately investigate gut microbiome alterations induced by HSD intervention, fecal samples from all experimental groups underwent metagenomic sequencing. Due to inherent genetic differences between Wistar and Dahl salt-sensitive rat strains, analyses were performed separately to avoid confounding effects: comparisons were made between WC and WH groups, and between DC and DH groups. The species composition results from the fecal samples are shown in Figure 6. Almost all detected species are bacteria, which provides a basis for our subsequent metagenomic analysis of gut microbiota.

#### 3.4.1 Gene composition and functional pathways

**Figure 7:**
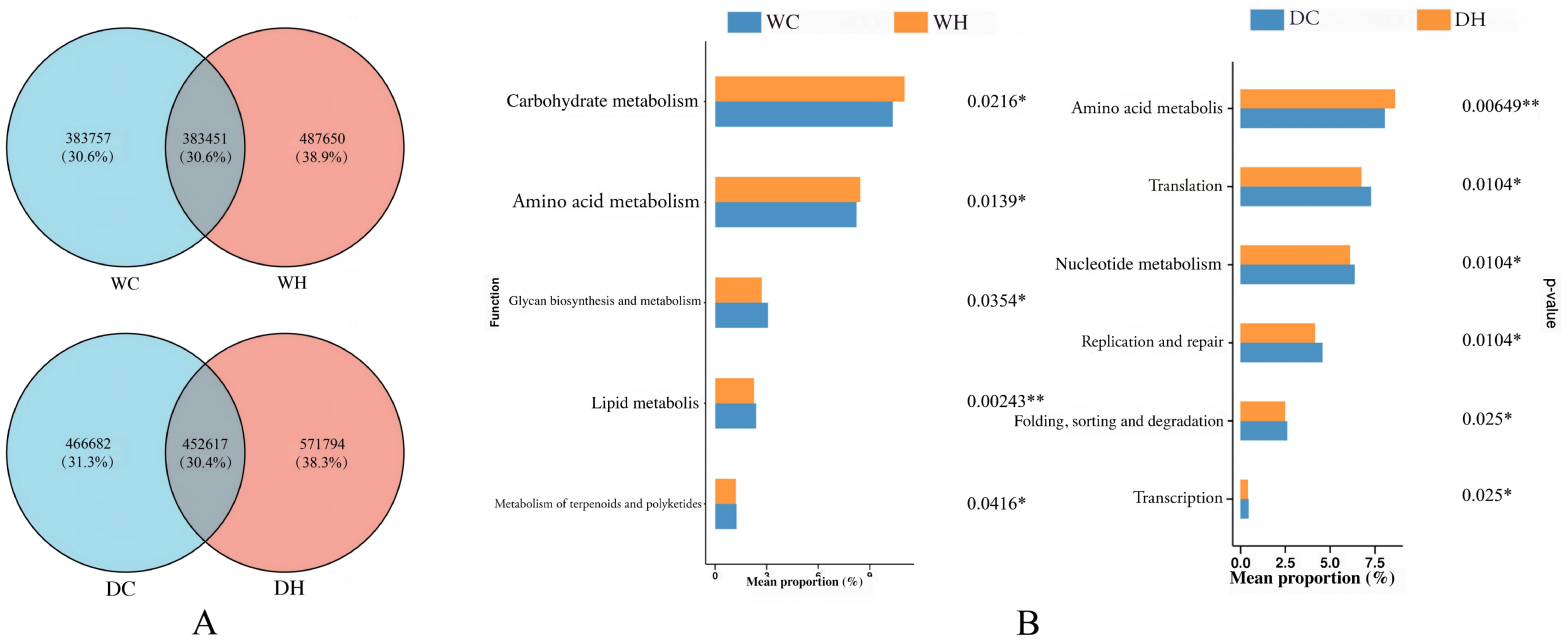
Microbial gene composition and functional enrichment. **(A)** Venn diagram showing the number of shared and unique non-redundant genes among groups. **(B)** KEGG pathway enrichment analysis of gut microbial genes. The left panel displays a bar plot of the mean abundance of functional categories; the *x*-axis indicates the mean proportion, and the *y*-axis shows the names of functional pathways. The right panel shows the corresponding *p*-values. The significance level was set at *p < 0.05, **p < 0.01.

To compare gut microbial gene content between control and HSD groups, intergroup differential abundance of genes was visualized (Figure 7A). The analysis revealed that only ~30% of non-redundant genes were shared between groups. This indicates that high-salt intake altered gut microbiota gene composition, consequently affecting both the enrichment of associated metabolic pathways and microbiota structure.

To analyze metabolic pathway changes resulting from gut microbial gene differences across groups, all genes were annotated to the KEGG database, and significantly enriched KEGG pathways (*p* < 0.05) were screened (Figure 7B, Supplementary Figure S1). KEGG pathway enrichment analysis revealed that compared with the WC group, the WH group showed significantly increased enrichment of gut microbial genes in carbohydrate metabolism and amino acid metabolism pathways (*p* < 0.05), significantly decreased enrichment in glycine biosynthesis, terpenoid and polyketide metabolism pathways (*p* < 0.05), and more pronounced decreased enrichment in lipid metabolism pathways (*p* < 0.01). Compared with the DC group, the DH group demonstrated significantly increased enrichment in amino acid metabolism pathways (*p* < 0.01), and significantly decreased enrichment in translation, nucleotide metabolism, replication and repair, folding/sorting/degradation, and transcription pathways (*p* < 0.05). In summary, HSD-induced gut microbial gene differences led to significant metabolic pathway variations between control and HSD groups, particularly in lipid and amino acid metabolism pathways.

#### 3.4.2 Microbial community structure

**Figure 8:**
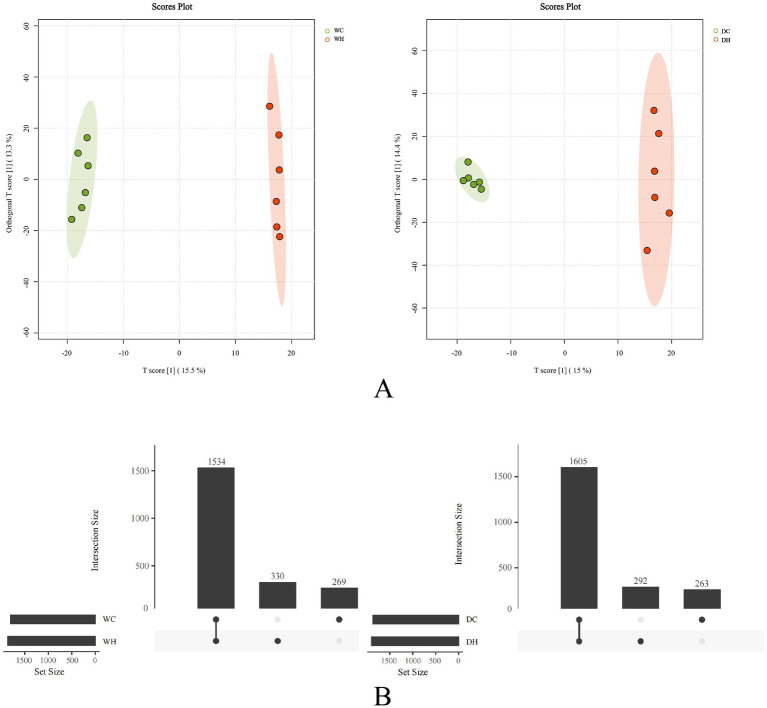
Microbial community structure and intergroup disparities. **(A)** OPLS-DA score plot of gut microbial profiles. Each point represents an individual sample, with green and red dots indicating the control and HSD groups, respectively. The two components (*x*- and *y*-axes) explain the maximum variance between groups, with percentages indicating the contribution of each component to the overall model. **(B)** Analysis of intergroup differences. The bar chart shows the number of microbial species per group. The points and connecting lines below represent set intersections and the number of shared species.

Orthogonal Partial Least Squares Discriminant Analysis (OPLS-DA) is a supervised discriminant analysis method that decomposes data into predictive and orthogonal components through partial least squares regression with orthogonal signal correction. This approach enhances model interpretability by effectively separating biological signals from noise, making it suitable for complex datasets. As shown in Figure 8A, OPLS-DA revealed distinct clustering separation between control and HSD groups, indicating significant alterations in gut microbiota composition and reduced richness/diversity induced by high-salt intake.

As shown in Figure 8B, among all detected gut microbiota in Wistar rats, 269 species were exclusive to the WC group, while 330 species were exclusive to the WH group. Among all detected gut microbiota in Dahl salt-sensitive rats, 263 species were exclusive to the DC group, while 292 species were exclusive to the DH group. This ~15% compositional divergence between control and high-salt groups demonstrates that HSD alters gut microbial diversity.

#### 3.4.3 Alpha diversity and F/B ratio

**Figure 9:**
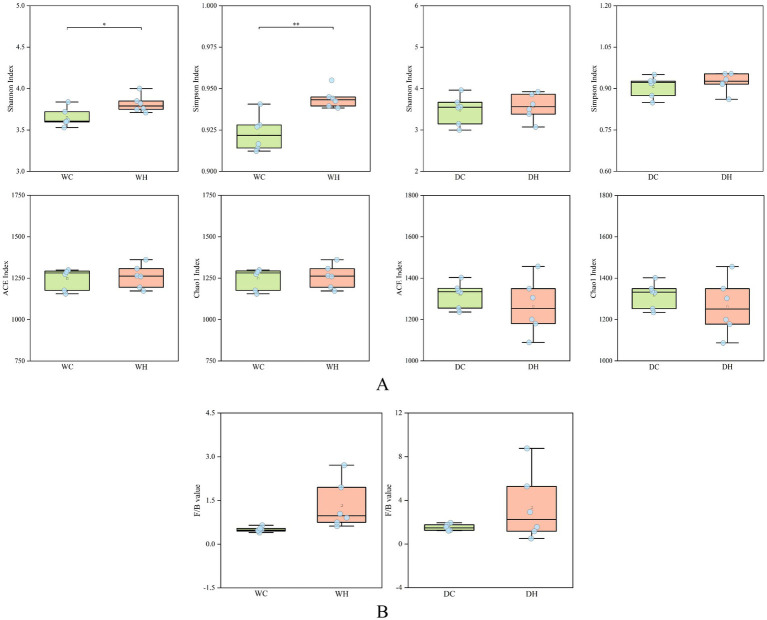
Alpha diversity and F/B ratio. **(A)** Alpha diversity analysis of the gut microbiota. **(B)** The F/B ratio in each group of rats. *p < 0.05, **p < 0.01.

Alpha diversity indices (Shannon, Simpson, ACE, and Chao1) are established metrics for assessing gut microbial diversity and richness. The Shannon and Simpson indices measure species diversity, reflecting both richness (number of species) and evenness (relative abundance distribution). Higher Shannon and Simpson values indicate increased community evenness but decreased richness. Meanwhile, ACE and Chao1 indices quantify species richness (absolute number of microbial taxa), where elevated values denote higher richness. As shown in Figure 9A and Supplementary Table S6, the WH group versus WC group: ACE and Chao1 indices showed no significant difference, while Shannon (*p* < 0.05) and Simpson (*p* < 0.01) indices significantly increased, indicating enhanced diversity. The DH group versus DC group: Shannon and Simpson indices remained unchanged, but ACE and Chao1 indices exhibited a decreasing trend, suggesting reduced richness. The above results show that HSD not only alters gut microbial diversity but also reduces richness in rats.

Over 30 bacterial phyla have been identified in the human gut microbiota, with their abundance and compositional changes closely linked to human health and disease. *Firmicutes* and *Bacteroidetes* constitute the majority of gut microbes. Post-infancy (>7 years), the *Firmicutes/Bacteroidetes* (F/B) ratio typically stabilizes, while its dysregulation is associated with metabolic disorders, including obesity and diabetes. As these two phyla comprise the majority of gut microbiota, shifts in their relative abundance reflect changes in dominant microbial species.

As shown in Figure 9B, HSD increased the F/B ratio in both Wistar and Dahl salt-sensitive rats. This indicates that high salt intake alters microbial richness, disrupts dominant phyla proportions, and induces gut dysbiosis.

#### 3.4.4 Taxon-specific alterations

**Figure 10:**
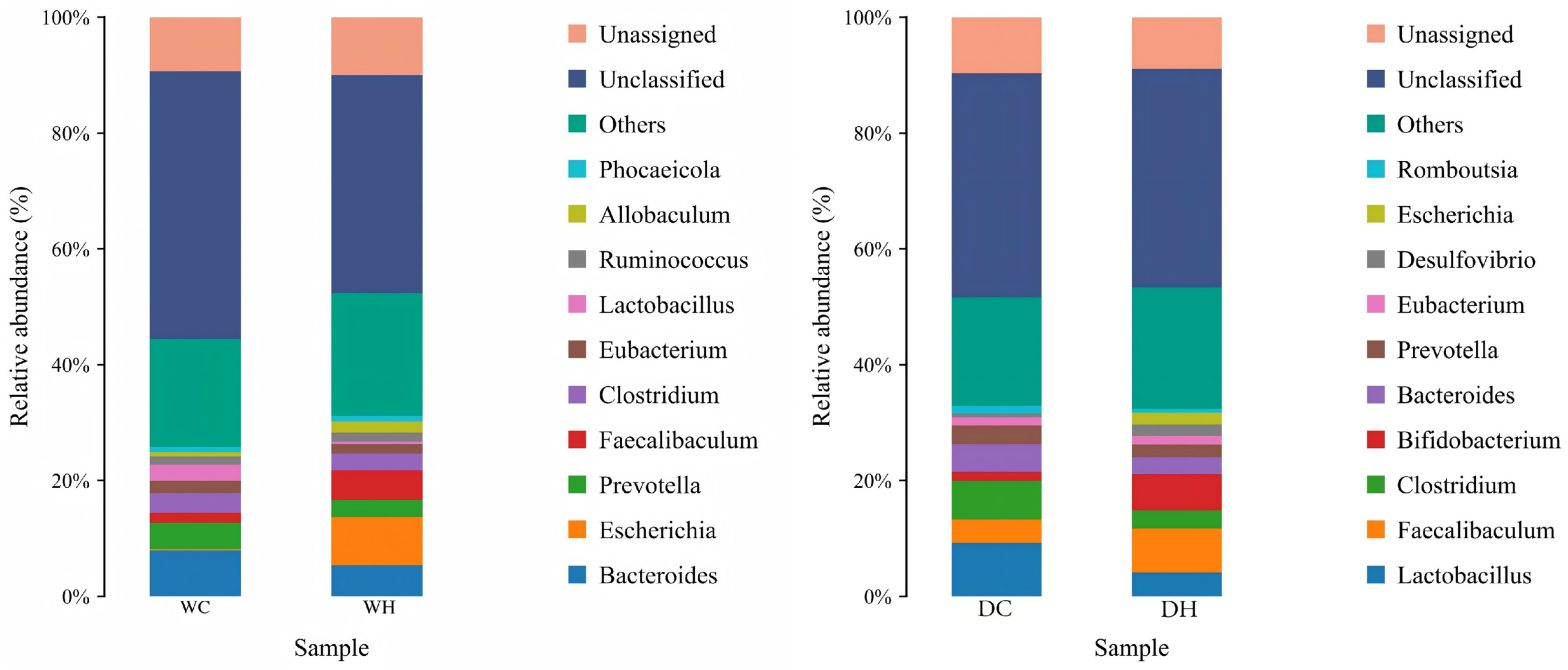
Taxonomic composition of gut microbiota across experimental groups. The bar chart illustrates the relative abundance of bacterial taxa at the genus level. The *x*-axis represents the experimental groups, and the *y*-axis indicates the relative abundance (percentage). Each bar is color-coded to represent a distinct bacterial genus, and the height of each segment corresponds to its proportional abundance within the group.

To further analyze the intergroup differences in gut microbiota, we identified the top 10 most abundant genera and visualized their relative abundance distribution in bar charts (Figure 10, Supplementary Tables S7, S8 and Figure S2).

In Wistar rats, compared with the WC group, the WH group exhibited decreased abundance of *Bacteroides*, *Prevotella*, *Clostridium*, *Eubacterium*, *Lactobacillus*, and *Phocaeicola*, but increased abundance of *Escherichia*, *Faecalibaculum*, *Ruminococcus*, and *Allobaculum* (*Faecalibaculum* significantly increased, *p* < 0.05).

In Dahl salt-sensitive rats, the DH group versus DC group displayed reduced abundance of *Lactobacillus*, *Clostridium*, *Bacteroides*, *Prevotella*, and *Romboutsia* (*Clostridium* significantly decreased, *p* < 0.05), alongside increased abundance of *Faecalibaculum*, *Bifidobacterium*, *Eubacterium*, *Desulfovibrio*, and *Escherichia* (*Bifidobacterium* significantly increased, *p* < 0.01).

In summary, HSD intervention altered the abundance of multiple bacterial genera, with particularly significant changes in *Faecalibaculum*, *Clostridium*, and *Bifidobacterium*.

### 3.5 Microbiota-inflammation correlations

**Figure 11:**
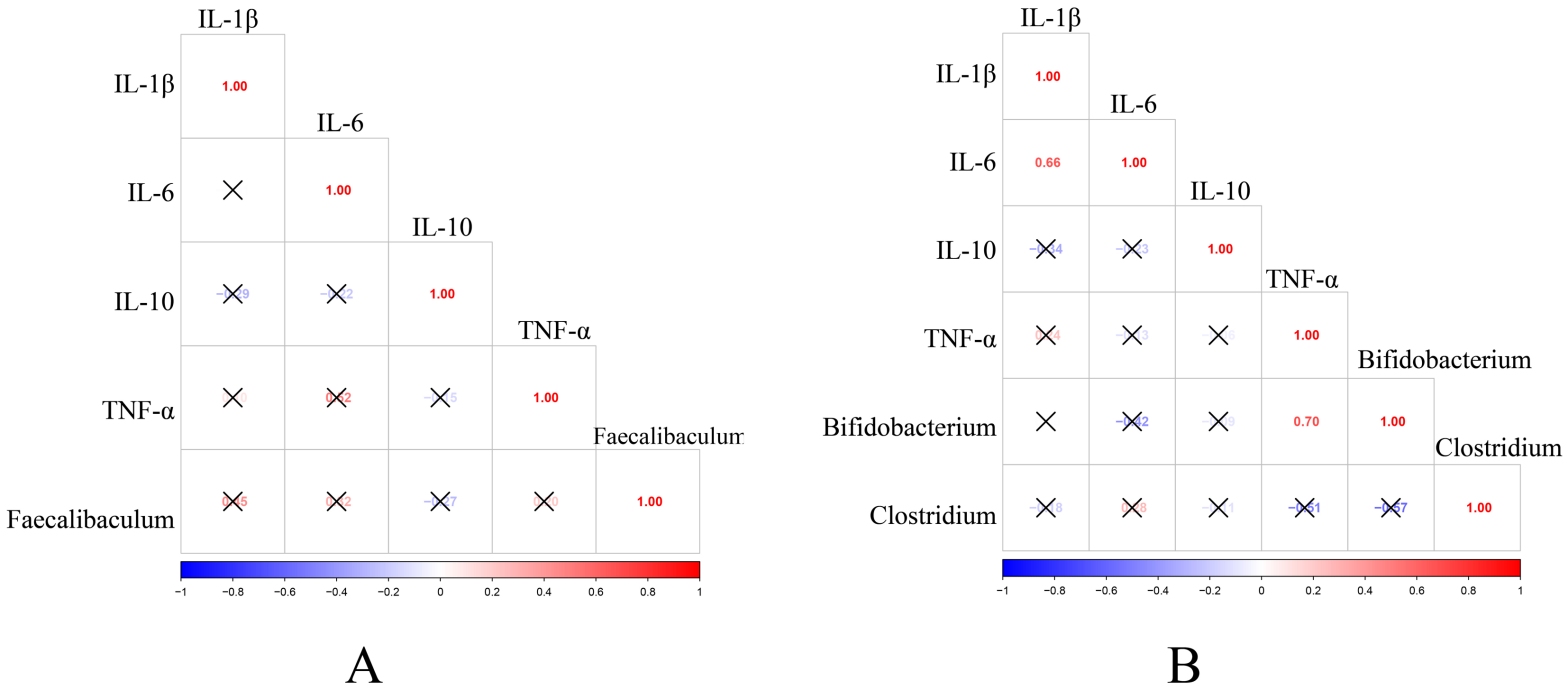
Correlation analysis between inflammatory cytokines and gut microbiota. **(A)** Wistar rats. **(B)** Dahl salt-sensitive rats. Shades of red represent positive correlations, while shades of blue represent negative correlations. An “×” denotes a statistically non-significant correlation (*p* > 0.05).

Following the confirmation that HSD affects both inflammatory cytokines and gut microbiota, Pearson correlation analysis was performed to investigate potential associations between IL-1β, IL-6, IL-10, and TNF-α levels and significantly altered gut microbiota in high-salt groups. As shown in Figure 11, no significant correlations were observed between inflammatory cytokines and gut microbiota in Wistar rats (*p* > 0.05). However, in Dahl salt-sensitive rats, IL-1β levels showed a significant positive correlation with IL-6 levels (*p* < 0.05), while TNF-α levels exhibited a significant positive correlation with *Bifidobacterium* abundance (*p* < 0.05).

These findings indicate significant correlations between inflammatory cytokine levels and gut microbiota abundance in Dahl salt-sensitive rats under high-salt intervention, but not in Wistar rats. This suggests that Dahl salt-sensitive rats not only respond to HSD through different pathways compared to Wistar rats, but their salt-sensitive phenotype further amplifies the impact on gut microbial ecology.

## 4 Discussion

In recent years, the chronic health hazards associated with HSD have become a focus of scientific research (He et al., 2020; Stanzione et al., 2023). Building on previous studies, this experiment aimed to investigate how salt sensitivity modifies the effects of high salt intake and to explore the mechanisms by which high salt intake induces various diseases through alterations in gut microbiota. By identifying the specific microbial and metabolic signatures associated with the salt-sensitive phenotype, our study not only provides a new perspective on diet-host-microbiota crosstalk but also offers a rationale for targeted, personalized dietary interventions for salt-sensitive individuals.

The Wistar rat is one of the most commonly used laboratory rat strains. Dahl salt-sensitive rats were derived from the Wistar strain and are now widely employed in animal studies. While direct extrapolation from animal models to humans should be approached with caution, the specific microbiota–immune interaction mechanisms identified in the salt-sensitive rats provide a strong theoretical basis and research direction for identifying potential biomarkers and therapeutic targets in human populations, thereby offering valuable insights for future clinical translation. At the same time, this approach provides a preclinical analogue for the differences in the effects of a HSD on the general population and salt-sensitive individuals in this way, which is why we did not compare salt-resistant rats with salt-sensitive rats. Therefore, this study utilized both Wistar and Dahl salt-sensitive rats to compare the specific effects of salt sensitivity under high-salt conditions. It should be noted that only male animals were used in this study to minimize the potential confounding effects of estrogen (Baker et al., 2017; Braniste et al., 2014; Wang et al., 2022; Acharya et al., 2019). A notable observation was that HSD led to increased body weight in Wistar rats but decreased body weight in Dahl salt-sensitive rats, indicating that salt sensitivity exacerbates the detrimental physiological effects of high salt intake, resulting in weight loss (Bier et al., 2018).

Regarding inflammatory cytokines, the WH group exhibited significantly higher IL-1β levels and lower IL-10 levels compared to the WC group. Given that IL-1β promotes inflammatory and immune responses while IL-10 suppresses these processes (Wang et al., 2023; Kong et al., 2023), these results indicate the presence of inflammatory disruption in WH rats. Although no significant changes were observed in interleukins in the DH group, a notable increase in TNF-α levels also suggests inflammatory activation (Li et al., 2023; Zhang et al., 2022). This perspective is further supported by colon pathological sections, which revealed substantial damage to colonic cells and tissue structure in high-salt groups (Xia et al., 2017; Guo et al., 2019; Hu et al., 2026; Peng et al., 2024; Zha et al., 2024), particularly in the DH group (Li et al., 2025; Fan et al., 2025; Fan et al., 2025). The more severe intestinal damage observed in the DH group is likely attributable to the uniquely potent role of TNF-α in directly disrupting the epithelial barrier and inducing apoptosis, a mechanism distinct from the inflammation mediated by IL-1β and IL-6 (Bradley, 2008; Hosic et al., 2020; Gao et al., 2025).

Under physiological conditions, the gut microbiota maintains a dynamic equilibrium. It undergoes gradual adaptations in response to minor disturbances, tending to establish a new homeostatic state—a process underlying its role in modulating human health and contributing to disease development (Zhao et al., 2018). A study evaluating the impact of HSD on murine gut microbiota revealed that, compared with controls, mice fed the HSD exhibited reduced abundance of beneficial bacteria such as *Lactobacillus*, along with increased Th17 cells and decreased regulatory T cells (Tregs). Subsequent probiotic supplementation with *Lactobacillus* reversed these changes, reducing Th17 and increasing Treg populations (Wilck et al., 2017a,b; Wei et al., 2017). These findings demonstrate that high-salt intake disrupts microbial homeostasis, promotes inflammatory responses, and reduces *Lactobacillus* abundance (Nagase et al., 2020). Furthermore, HSD compromises microbial richness and diversity while concurrently activating inflammatory pathways (Ye et al., 2022; Wang et al., 2022). Gut microbiota dysregulation has been identified as one mechanism contributing to systemic inflammation, oxidative stress, and vascular injury in non-alcoholic fatty liver disease (Li et al., 2022). Under normal conditions, however, the gut microbiota helps mitigate lipid accumulation and prevent cardiovascular disease by modulating peripheral ketone body levels—effects attributed to beneficial genera such as *Lactobacillus* involved in lipid metabolism and regulation of unsaturated fatty acid and ketone synthesis and degradation (Zhai et al., 2022; Pokharel et al., 2023).

Our results revealed that only approximately 30% of non-redundant genes were shared between the control and high-salt groups, accompanied by about a 15% difference in microbial composition, indicating that the HSD altered gut microbiota homeostasis. To move from a descriptive account of dysbiosis to a functional one, we compared the enriched KEGG pathways across experimental groups, which revealed how the structural remodeling of the microbiota impacted its metabolic potential. Compared with the WC group, the WH group showed significantly reduced enrichment of microbial genes in lipid metabolism pathways, suggesting a decreased capacity for metabolizing lipids such as unsaturated fatty acids and ketone bodies, thereby increasing the risk of diseases such as atherosclerosis and fatty liver disease. In contrast, the DH group exhibited significantly increased enrichment in amino acid metabolism pathways compared to the DC group. Since amino acids serve as precursors for secondary bile acid synthesis, this implies that high salt intake may exert systemic effects by altering the composition and concentration of bile acids (Zeng et al., 2024). Alpha diversity analysis indicated reduced richness but increased diversity in the high-salt groups, manifested as an elevated F/B ratio (Tian et al., 2025). The observed increase in the F/B ratio under HSD is a crucial alteration. In our study, this ratio increase co-occurred with elevated systemic pro-inflammatory cytokines and colonic damage, strongly suggesting it contributes to the detrimental, rather than adaptive, host response (Canyelles et al., 2023). This finding finds resonance in human studies; for instance, an elevated F/B ratio has been consistently reported in cohorts of hypertensive (Tsiavos et al., 2024; Cai et al., 2023). The consistency between our model and human data underscores the F/B ratio as a conserved microbial feature of salt-related pathophysiology and reinforces the translational value of our experimental findings. Further taxonomic analysis revealed a significant increase in *Enterobacter* in the WH group, and a significant increase in *Bifidobacterium*, accompanied by a decrease in *Clostridium* in the DH group (Zheng et al., 2023a,b). Although not statistically significant, *Lactobacillus* showed a decreasing trend in both high-salt groups, suggesting a heightened risk of inflammatory responses (Do et al., 2022). Both Wistar and Dahl salt-sensitive rats exhibited gut microbiota dysbiosis following high-salt exposure, including increased inflammatory cytokines, colonic tissue injury, and alterations in microbial richness and composition. These findings indicate that high-salt intake itself acts as a strong perturbing factor, exerting detrimental effects across different genetic backgrounds. However, the DH groups displayed more severe colonic pathological injury and a greater reduction in specific bacterial taxa (e.g., *Clostridium*) compared with the WH group, suggesting that salt sensitivity amplifies the deleterious impact of high-salt intake. In terms of microbial composition, *Faecalibaculum* was markedly increased in Wistar rats, whereas *Bifidobacterium* was predominantly elevated in salt-sensitive rats. This implies that hosts with distinct genetic backgrounds may adopt different adaptive or pathological remodeling strategies in response to the same environmental stressor. At the metabolic pathway level, Wistar rats mainly showed downregulation of lipid metabolism (Xu et al., 2025; Liu et al., 2024; Boldarine et al., 2020), indicating that it may be related to the risk of cardiovascular diseases such as atherosclerosis. Salt-sensitive rats exhibited significant upregulation of amino acid metabolism, which was linked to secondary bile acid biosynthesis (Carvalho et al., 2024; Xu et al., 2023). This suggests that salt sensitivity may confer health risks through distinct metabolic axes.

*Enterobacter*, which includes opportunistic pathogens such as *E. coli*, may increase the risk of intestinal infections, diarrhea, and dysentery when enriched under high-salt conditions (Sun et al., 2020). *Clostridium*, a beneficial gut genus, alleviates inflammation and allergic reactions by activating intestinal epithelial cells, enhancing barrier function, and modulating immune activity (Guo et al., 2020). *Bifidobacterium*—like *Lactobacillus* and *Clostridium* arebeneficial bacteria used to treat diarrhea, constipation, and functional dyspepsia related to dysbiosis (Bertuccioli et al., 2025). The significant increase in *Bifidobacterium* may represent a compensatory response to offset immune risks associated with reduced *Clostridium* abundance. This shift may reflect a selective expansion of *Bifidobacterium* strains resilient to the high-salt and pro-inflammatory environment (Zheng et al., 2023a,b; Gandhi & Shah, 2016; Robles-Vera et al., 2020; Veselovsky et al., 2020), particularly as *Bifidobacterium* levels correlated positively with TNF-α in the DH group (Balan et al., 2022). Studies have shown that Th17 and Treg cell activities are influenced by the gut microbiota (Aguiar et al., 2018; Kleinewietfeld et al., 2013; Wu et al., 2013). *Enterobacter*, *Clostridium*, *Bifidobacterium*, and *Lactobacillus* collectively regulate intestinal immune responses via the Th17/Treg axis, suggesting that the gut microbiota represents a potential therapeutic target for mitigating disease risks induced by HSD (Pang et al., 2020). Our previous work has demonstrated that high-salt intake elevates blood pressure, impairs vascular endothelial function, and disrupts bile acid metabolism (Li et al., 2022). In the current study, we further observed that changes in body weight and systemic inflammatory cytokine levels represent reliable phenotypic indicators of high-salt–induced tissue injury and predictors of future metabolic and cardiovascular risk. Future studies will aim to integrate multi-system physiological parameters to provide a more comprehensive understanding of the systemic effects of high-salt intake.

The findings of this study provide several important insights into the adverse health impacts of high-salt intake in humans. First, the results suggest that individual salt sensitivity may represent a key determinant of dietary risk and should be given greater consideration in clinical nutritional assessment and personalized dietary guidance. Second, the identified microbial taxa (e.g., *Clostridium*, *Bifidobacterium*) and metabolic pathways (e.g., bile acid metabolism) may serve as potential biomarkers for evaluating high-salt exposure or the efficacy of interventions in human populations. Finally, targeted modulation of the gut microbiota through probiotic or microbiome-directed nutritional approaches may offer a novel strategy to protect salt-sensitive individuals from the detrimental effects of HSD. Notably, the translational relevance of our findings is supported by independent clinical data. A human study investigating the immune response to HSD (GEO: GSE265934) reported congruent microbial and immunological shifts, including an increase in *Bifidobacterium* abundance, elevated Th17 cell levels, and heightened gut permeability, which were associated with disease pathogenesis (Sharma et al., 2024). This consistency across species underscores the gut microbiota as a conserved player in salt-sensitive physiological disturbances and strengthens the potential translational impact of our mechanistic findings.

Looking forward, the correlative nature of this study underscores the necessity for future mechanistic investigations to establish causality. Specifically, fecal microbiota transplantation experiments are required to confirm whether the microbiota from salt-sensitive hosts is sufficient to transmit the phenotypic vulnerability. Furthermore, targeted interventional studies, such as administering probiotic strains or implementing personalized dietary salt modulation, are essential next steps to validate the therapeutic potential of manipulating the gut ecosystem.

## 5 Conclusion

This study comparatively analyzed the detrimental effects of the HSD on both general and salt-sensitive constitutions. Although the resulting damage and underlying mechanisms are similar between the two models, the key metabolites involved differ significantly, thus providing a theoretical basis for further targeted research. A limitation of this study is that neither Dahl salt-sensitive nor Wistar rats fully recapitulate human physiology. Nevertheless, our comparative metagenomic study demonstrates that host salt sensitivity is a pivotal factor shaping the gut microenvironment’s response to an HSD. It not only exacerbates the severity of microbial dysbiosis and intestinal damage but also steers the host-microbiota system toward distinct ecological and functional trajectories. The identified disparities in microbial consortia and metabolic pathways, coupled with their correlations to inflammatory markers, provide a novel mechanistic framework for understanding the heightened disease susceptibility in salt-sensitive individuals and highlight the gut microbiome as a promising target for future preventative strategies.

## Data Availability

The raw sequence data reported in this paper have been deposited in the Genome Sequence Archive (Zhang et al., 2025) in National Genomics Data Center (CNCB-NGDC Members and Partners, 2025), China National Center for Bioinformation/Beijing Institute of Genomics, Chinese Academy of Sciences (GSA: CRA035238) that are publicly accessible at https://ngdc.cncb.ac.cn/gsa.
