# Supplementary material for: Salt sensitivity potentiates high-salt diet-induced intestinal barrier disruption and gut microbiome dysbiosis in rats
Source: Front Microbiol. 2026 Jan 9;16:1718782. doi: 10.3389/fmicb.2025.1718782 (PMC12827649; doi:10.3389/fmicb.2025.1718782)
Supplement: Supplementary file 1 [file Data_Sheet_1.ZIP › Supplementary Files.docx]

**Table S1. The specific components of rat feed.**

| **The Specific Components of Rat Feed (Not Including the Content of NaCl )** | |
| --- | --- |
|  |  |
| **Components** | **Proportion** |
|  |  |
| Casein | 20 |
| Methionine | 0.3 |
| Sucrose | 50 |
| Corn Starch | 15 |
| Cellulose | 5 |
| Corn Oil | 5 |
| Multi Mineral S10001 | 3.5 |
| Multidimensional V10001 | 1 |
| Choline Bitartrate | 0.2 |
| Total | 100 |

| **Table S2. Analytical performance parameters of ELISA kits** | | | | | |
| --- | --- | --- | --- | --- | --- |
|  | Catalog No | Intra-assay CV (%) | Inter-assay CV (%) | Sensitivity (pg/mL) | Detection Range (pg/mL) |
|  |  |  |  |  |  |
| TNF-α | E-EL-R2856 | < 10 | < 10 | 9.38 | 15.63 - 1000 |
| IL-6 | E-EL-R0015 | < 10 | < 10 | 7.5 | 12.5 - 800 |
| IL-1β | E-EL-R0012 | < 10 | < 10 | 18.75 | 31.25 - 2000 |
| IL-10 | E-EL-R0016 | < 10 | < 10 | 18.75 | 31.25 - 2000 |

| **Table S3. Basic sequencing and assembly statistics for individual samples** | | | | |
| --- | --- | --- | --- | --- |
| Sample ID | Raw Data (bp) | Approx. Raw Data (Gb) | Contig Num. | Mapped(%) |
|  |  |  |  |  |
| WN-1 | 6391069210 | 6.39 | 216443 | 91.54 |
| WN-2 | 7589715930 | 7.59 | 249606 | 92.12 |
| WN-3 | 6902632328 | 6.9 | 231196 | 91.05 |
| WN-4 | 6075600902 | 6.08 | 202001 | 90.05 |
| WN-5 | 6877983694 | 6.88 | 216511 | 91.25 |
| WN-6 | 6473450398 | 6.47 | 274662 | 88.1 |
| WH-1 | 6259859916 | 6.26 | 240024 | 90.48 |
| WH-2 | 6194775682 | 6.19 | 238173 | 89.28 |
| WH-3 | 6719198306 | 6.72 | 210849 | 88.62 |
| WH-4 | 7078751572 | 7.08 | 208167 | 91.5 |
| WH-5 | 6899576410 | 6.9 | 152011 | 94.12 |
| WH-6 | 6867465302 | 6.87 | 187551 | 90.75 |
| DC-1 | 6728272640 | 6.73 | 242866 | 94.31 |
| DC-2 | 6899685886 | 6.9 | 298082 | 91.08 |
| DC-3 | 6658226938 | 6.66 | 309895 | 89.33 |
| DC-4 | 6184942364 | 6.18 | 222913 | 92.77 |
| DC-5 | 6853905412 | 6.85 | 283611 | 91.68 |
| DC-6 | 6608491926 | 6.61 | 258029 | 92.92 |
| DH-1 | 6271139916 | 6.27 | 160872 | 95.08 |
| DH-2 | 6381285444 | 6.38 | 205083 | 93.6 |
| DH-3 | 6601694834 | 6.6 | 251604 | 93.95 |
| DH-4 | 6604057140 | 6.6 | 259495 | 93.57 |
| DH-5 | 6119407318 | 6.12 | 268327 | 93.19 |
| DH-6 | 6170190446 | 6.17 | 153597 | 95.3 |

**Table S4. Changes in body weight of rats throughout the modeling period.**

|  | WC group  (M±SD) | WH group  (M±SD) | DC group  (M±SD) | DH group  (M±SD) |
| --- | --- | --- | --- | --- |
| Baseline (g) | 308±16 | 306±8 | 233±10 | 232±13 |
| 2^nd^ week (g) | 358±25 | 350±10 | 294±9 | 274±19 |
| 4^th^ week (g) | 351±24 | 351±13 | 293±8 | 278±23 |
| 6^th^ week (g) | 346±27 | 355±7 | 291±10 | 283±25 |
| 8^th^ week (g) | 330±25 | 350±10 | 285±9 | 279±22 |

**Table S5. Inflammatory cytokine levels of rats in each group.**

|  | WC group  (M±SD) | WH group  (M±SD) | DC group  (M±SD) | DH group  (M±SD) |
| --- | --- | --- | --- | --- |
| IL-1β (pg/mL) | 25.6±4.06 | 31.01±4.24 | 27.43±14.18 | 28.58±0.94 |
| IL-6 (pg/mL) | 10.52±0.77 | 11.23±0.4 | 10.06±0.31 | 9.84±0.17 |
| IL-10 (pg/mL) | 10.32±2.28 | 6.2±2.94 | 14.18±6.29 | 14.14±4.2 |
| TNF-α (pg/mL) | 25.35±12.55 | 28.48±5.11 | 16.02±1.89 | 26.04±3.88 |

**Table S6. Alpha diversity indices of rats in each group.**

|  | WC group  (M±SD) | WH group  (M±SD) | DC group  (M±SD) | DH group  (M±SD) |
| --- | --- | --- | --- | --- |
| Shannon Index | 3.65±0.11 | 3.82±0.10 | 3.48±0.35 | 3.56±0.32 |
| Simpson Index | 0.92±0.01 | 0.94±0.01 | 0.91±0.04 | 0.92±0.03 |
| ACE Index | 1247.80±64.18 | 1260.41±69.96 | 1319.20±62.30 | 1263.87±132.55 |
| Chao1 Index | 1247.63±64.34 | 1260.21±69.73 | 1377.33±62.72 | 1262.03±133.14 |

**Table S7. Composition of the predominant gut microbiota in Wistar rats.**

| **Name** | **Category** | **Mean reads**  **(Control group)** | **Mean reads**  **(High-salt group)** |
| --- | --- | --- | --- |
| *Bacteroides* | G- | 419883.17 | 273760.50 |
| *Escherichia* | G- | 8581.83 | 470226.17 |
| *Prevotella* | G- | 244618.83 | 154266.50 |
| *Faecalibaculum* | G- | 93583.83 | 265075.17* |
| *Clostridium* | G+ | 180139.50 | 145796.67 |
| *Eubacterium* | G+ | 115688.67 | 89145.00 |
| *Lactobacillus* | G+ | 153909.17 | 20828.67 |
| *Ruminococcus* | G+ | 76231.50 | 78416.17 |
| *Allobaculum* | G- | 35008.83 | 101322.67 |
| *Phocaeicola* | G- | 50227.33 | 48871.67 |

The abbreviations G- (Gram-negative) and G+ (Gram-positive) are used throughout this table. The significance level was set at **p* < 0.05.

**Table S8. Composition of the predominant gut microbiota in Dahl salt-sensitive rats.**

| **Name** | **Category** | **Mean reads (Control group)** | **Mean reads (High-salt group)** |
| --- | --- | --- | --- |
| *Lactobacillus* | G+ | 467110.00 | 213084.83 |
| *Faecalibaculum* | G- | 217280.83 | 394750.83 |
| *Clostridium* | G+ | 342364.00 | 163325.50* |
| *Bifidobacterium* | G+ | 79531.17 | 326972.33** |
| *Bacteroides* | G- | 254069.83 | 142987.00 |
| *Prevotella* | G- | 165516.83 | 114273.33 |
| *Eubacterium* | G+ | 73378.50 | 76188.83 |
| *Desulfovibrio* | G- | 31616.83 | 105748.33 |
| *Escherichia* | G- | 1530.50 | 102456.33 |
| *Romboutsia* | G+ | 66569.33 | 36191.17 |

The abbreviations G- (Gram-negative) and G+ (Gram-positive) are used throughout this table. The significance level was set at **p* < 0.05 and ***p* < 0.01.


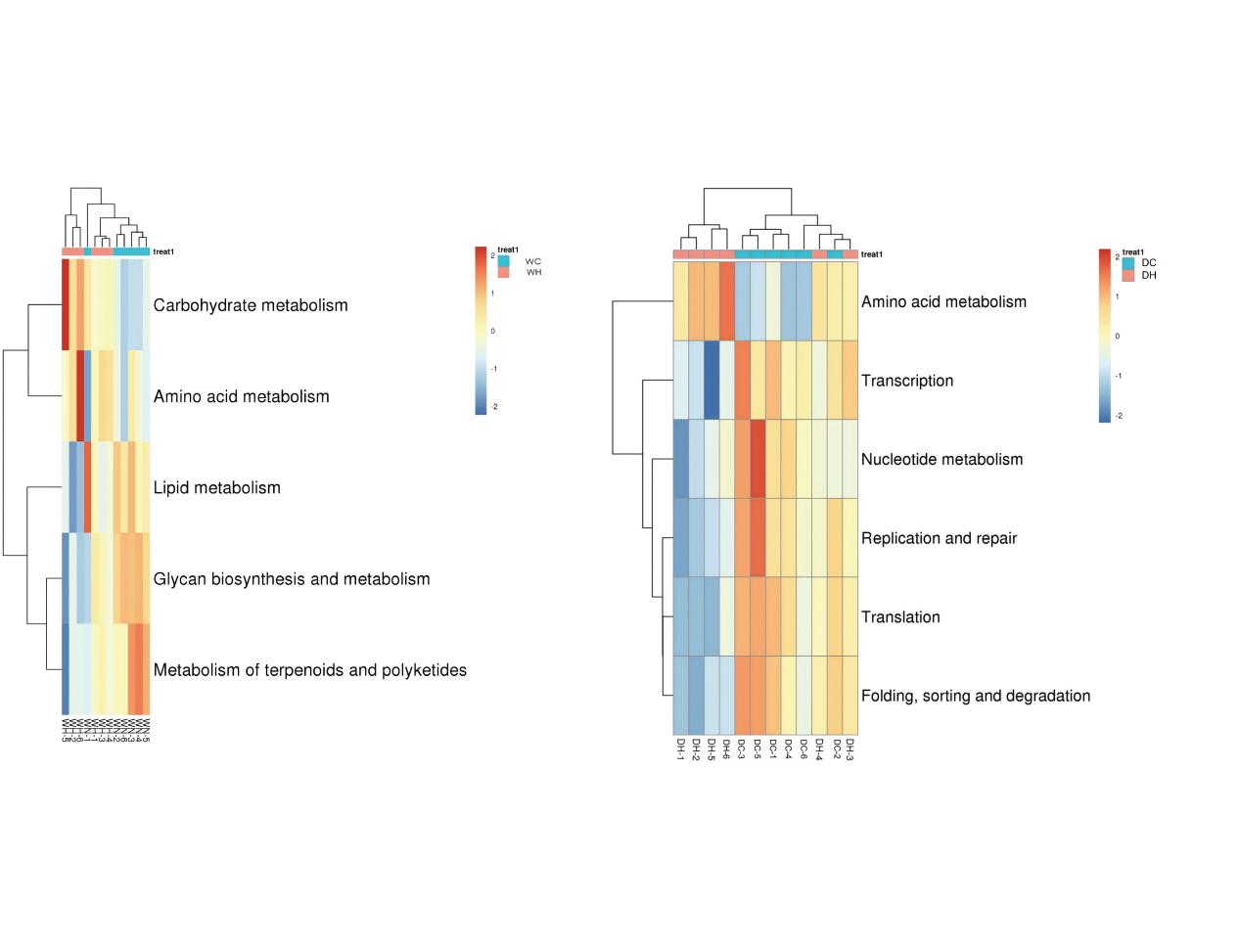


**Figure S1. The heatmap of KEGG pathway enrichment analysis of gut microbial genes..**


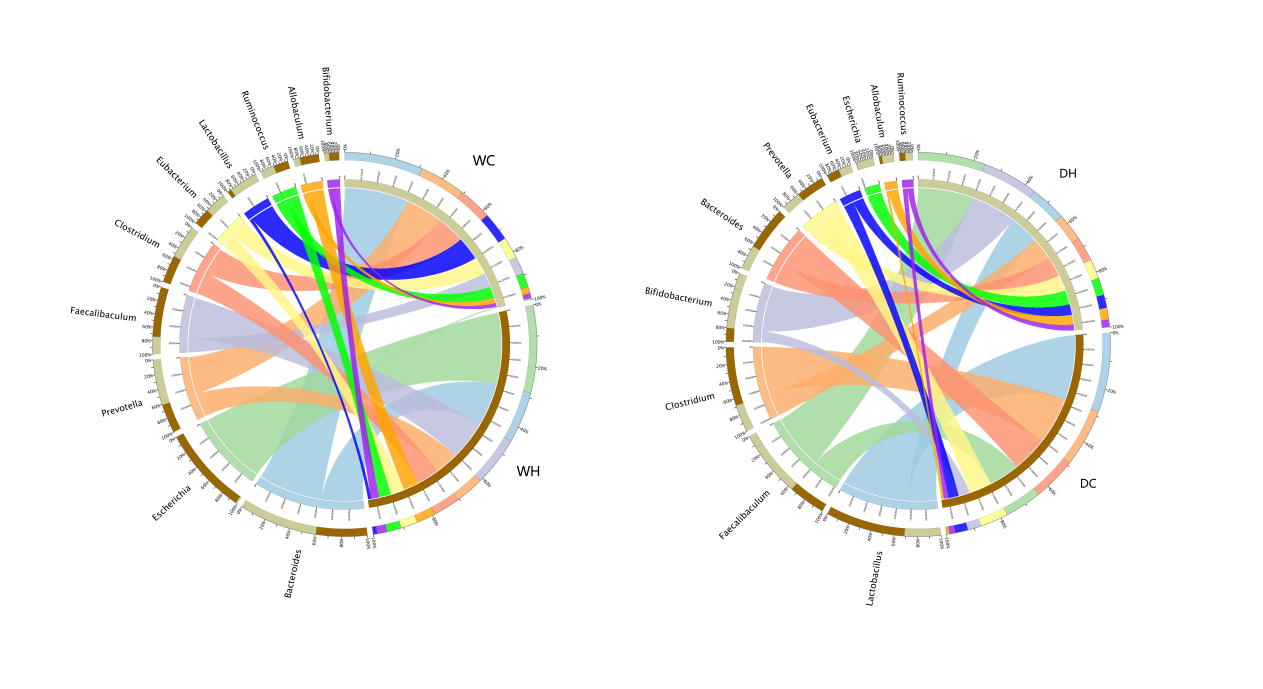


**Figure S2.** The right half of the outer circle represents the group, the left half represents the annotated gut microbiota type, and the scale represents the proportion of abundance.
