# Supplementary figures and images for: Salt sensitivity potentiates high-salt diet-induced intestinal barrier disruption and gut microbiome dysbiosis in rats
Source: Front Microbiol. 2026 Jan 9;16:1718782. doi: 10.3389/fmicb.2025.1718782 (PMC12827649; doi:10.3389/fmicb.2025.1718782)

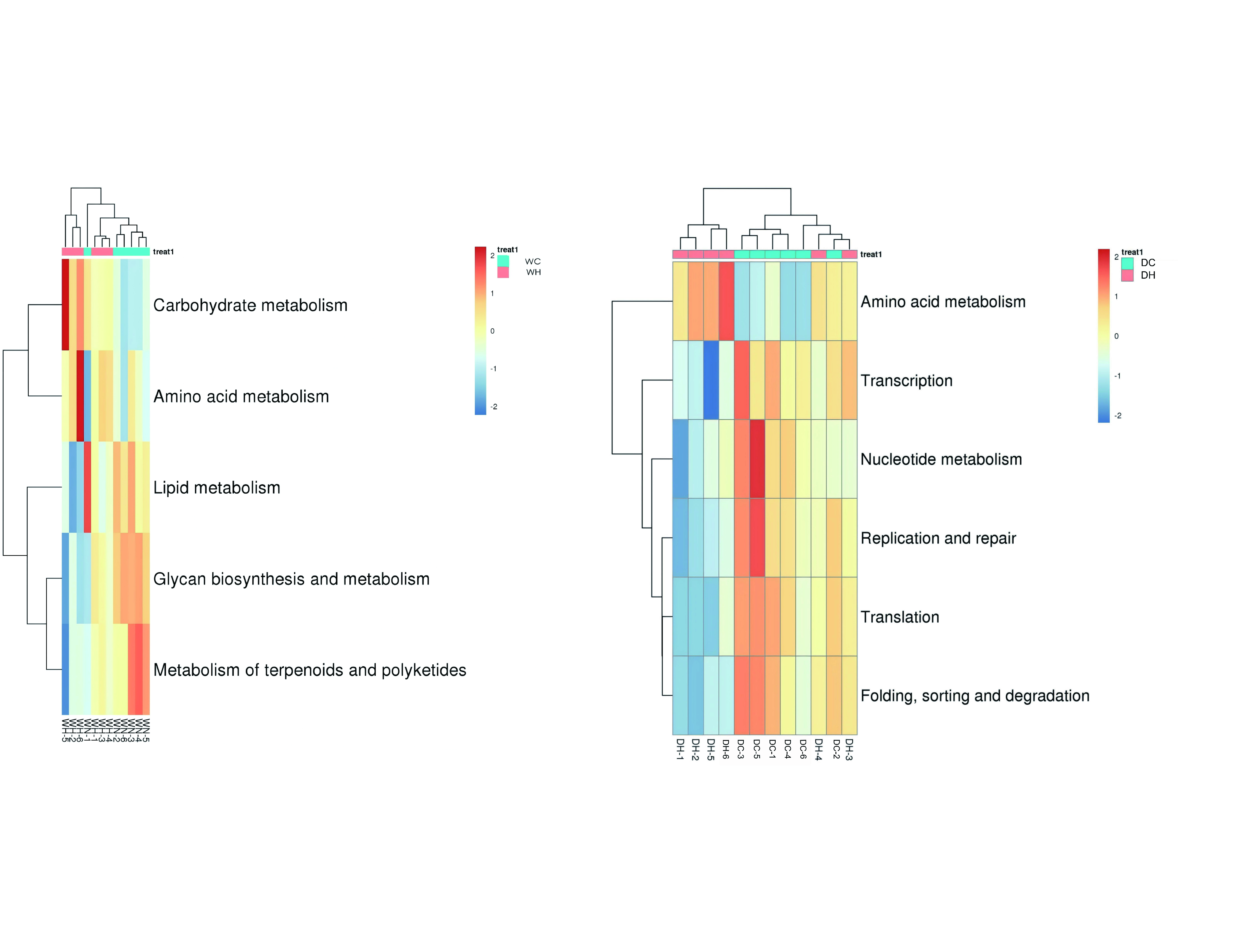

Supplement: Supplementary file 1 [file Data_Sheet_1.ZIP › Figure S1.jpg]
